# The role of civil society organizations in alcohol control during the COVID-19 pandemic across six countries in Africa

**DOI:** 10.1007/s44155-023-00049-x

**Published:** 2023-08-03

**Authors:** Kashish Aneja, Aadielah Maker Diedericks, Sam Halabi, Katie Gottschalk, Kerry Waddell, Juan E. Tello

**Affiliations:** 1 Georgetown University Law Center, Washington, USA; 2 Southern African Alcohol Policy Alliance—SAAPA, Johannesburg, South Africa; 3 McMaster Health Forum, McMaster University, Hamilton, Canada; 4 World Health Organization, Geneva, Switzerland

**Keywords:** Alcohol control, Civil society organizations, COVID-19 pandemic, Public awareness campaigns, Capacity building, Legal interventions

## Abstract

**Background:**

The differing global trends in alcohol consumption and policy measures implemented during the COVID-19 pandemic warrant a closer look at the actions taken by civil society organisations (CSOs) and community-led efforts to describe how they may influence and accelerate action for change in alcohol control measures. This paper analyses actions undertaken by CSOs at the national and local levels to safeguard communities and improve alcohol control policies during the COVID-19 pandemic in six African countries.

**Methods:**

A cross-sectional survey was distributed via email to CSOs involved in alcohol prevention, outreach and policy development in Kenya, Lesotho, Namibia, South Africa, Tanzania and Zimbabwe. Individuals (n = 19) working at CSOs responded to the questionnaire from February to March 2022. Questions related to the role of CSOs during the pandemic are analysed and synthesised in this paper. 19 CSOs respondents representing the six countries were included in the study.

**Results:**

Action areas led by CSOs during the COVID-19 pandemic included: (i) direct lobbying advocacy, (ii) conducting public awareness media campaigns and (iii) legal and regulatory interventions linked to the pandemic.

**Conclusions:**

Given the size of the challenges governments faced during the COVID-19 pandemic, the role of CSOs, during the ongoing pandemic and beyond, has become even more relevant to strengthen advocacy and public health interventions for alcohol control in Southern Africa. For this, CSOs should have a “seat at the table” when public health policies are designed, discussed and enforced.

## Introduction

The COVID-19 pandemic highlighted the extent to which alcohol consumption is closely intertwined with the mitigation efforts implemented to curb global transmissions during pandemic events. This includes measures such as stay-at-home directives that shifted consumption from on-premises to at-home, as well as closures of bars, pubs, and restaurants that increased off-premise sales through e-commerce and retail shops, among others [1]. During the COVID-19 pandemic, even a few heads of states such as the South African President recognised the importance of alcohol control measures [2, 3]. Research examining how COVID-19 alcohol policies have changed alcohol consumption patterns has mainly focused on Europe and North America [4–8]. The effects of the COVID-19 pandemic on alcohol consumption and regulation in Africa are less well known. The differing global trends in alcohol consumption and policy measures implemented during the COVID-19 pandemic warrant a closer look at the actions taken by civil society organizations (CSOs).

Alcohol consumption is a risk factor for developing non-communicable and communicable diseases, premature mortality, injury and domestic violence, all of which have severe economic and societal costs [9]. Alcohol consumption also weakens the immune system, making individuals more susceptible to infections and increasing the risk of severe illness, including COVID-19 [10, 11].

The fear of COVID-19 infection, uncertainties about the future, isolation, loneliness and social disruptions have significantly influenced global mental health [12]. Loneliness has been considered a risk factor for alcohol abuse [13]. The COVID-19 pandemic is estimated to have caused a 27.6% rise in cases of major depressive disorder and a 25.6% increase in cases of anxiety disorder worldwide, with low-and-middle-income countries bearing the brunt of the burden [14]. Countries in Africa have not been immune to this trend. A survey in South Africa noted that between July and November 2020, nearly 50% of participants self-reported consuming alcohol assimilable to heavy-episodic drinking, with most participants having purchased alcohol illegally despite the COVID-19 restrictions [12].

The challenges associated with alcohol consumption and the COVID-19 pandemic are enduring and warrant crosscutting, evidence-based solutions. Policies regulating the production, supply and purchasing of alcoholic beverages are effective and proven strategies for reducing alcohol consumption and COVID-19 transmission. However, in the wake of the COVID-19 pandemic, governments have sparsely taken up these possible interventions [1]. On the contrary, CSOs have filled these gaps and omissions; for example, by raising awareness about the potential harm caused by alcohol consumption to own health and to others or lobbying against industry interference in government decisions. Community-led efforts can be critical to creating momentum and accelerating action for change. The need for CSOs’ involvement to ensure synergies between prosocial behaviours and governance has been positioned and explained by the Lancet Commission on lessons for the future from the COVID-19 pandemic [15]. The Lancet Commission also calls for increased sustainable development funding from all sectors, including civil society. This paper analyzes actions undertaken by civil society organizations to safeguard communities and improve alcohol control policies during the COVID-19 pandemic in six African countries.

## Methods

A cross-sectional survey was distributed via email to CSOs involved in alcohol prevention, outreach and policy development in Kenya, Lesotho, Namibia, South Africa, Tanzania and Zimbabwe. Individuals working at non-governmental organizations and community-based organizations responded to the questionnaire from February to March 2022.

The Southern African Alcohol Policy Alliance (SAAPA) distributed the self-completion 23-open ended question survey to its members and, in turn, distributed it to their contacts via email. Respondents did not receive any compensation.

Three sets of open-ended questions were asked in the questionnaire. The first set related to the regulatory changes to alcohol from January 2020 to July 2021 (17 questions). The second set was related to industry interference and corporate social responsibility (3 questions). Finally, a third set related to the role of CSOs during the pandemic (3 questions). The responses to the third set of questions are reflected in this paper. Specifically, these questions referred to the role played by CSOs in alcohol control during the COVID-19 pandemic.

Overall, the response rate was 100% but not all 19 enquired CSOs responded to all three questions related to the role of CSOs during the pandemic. The interviewees disclosed no circumstances that could represent a potential conflict of interest concerning the scope, development or outcome of this work. While some of these CSOs focused their work generally on non-communicable disease risk factors regulation, some work specifically on alcohol control, and a few on specific measures such as road safety.

The responses to all the open questions were anonymized and analyzed. Two authors (KA and AMD) summarised and discussed the findings with the research group. The group collectively identified four areas of CSO intervention. The synthesis and analysis across CSOs were carried out using the four areas adopted (Annex 1). Different sources were used to triangulate the information, including a desk review of publications and blogs published by CSOs. Similar steps served to refine the discussion and conclusions.

WHO Publications Review Committee oversees and provides clearance for all documents related to public health emergencies, including the COVID-19 pandemic. The decision of the Committee is made at the planning and executive clearance stages to ensure relevance, consistency, methodological validity and compliance with quality standards. The Committee approved the protocol in the context of the broader study [1].

### Limitations

Limitations of the methods include the possible bias in selecting the CSOs interviewed and the validation of the questionnaire. The selection of CSOs may have been limited to a pre-existing network of CSOs. The number of CSOs working in the alcohol prevention field (denominator) is not necessarily known as there is no register to consult and some community-based organizations may work on alcohol only as a risk factor for non-communicable diseases or violence against women and children. Given the low number of CSOs, a group of experts working on advocacy, lobbying and public health designed and reviewed the questionnaire. During its administration, online support was provided to clarify and address specific challenges raised by the interviewed CSOs.

## Results

Among the 19 respondents, five action areas were led by CSOs during the COVID-19 pandemic: direct lobbying advocacy (in three of the six countries), conducting public awareness media campaigns (in five of the six countries); and legal and regulatory interventions (in South Africa). Other action areas led by CSOs included building capacity through community training (Namibia).

The countries and sample sizes are displayed in Table 1 below.

**Table 1:** Number of CSOs enquired and respondents by country

Country	Count
Lesotho	7
Namibia	5
South Africa	4
Kenya	1
Tanzania	1
Zimbabwe	1
Total	19

## Discussion

CSOs are instrumental in creating momentum and accelerating action. CSOs can play a role in setting priorities for national agendas, increasing public demand for policies, laws and regulations, and ensuring that services reach communities [15, 16]. CSOs in the six studied countries of Southern Africa played an important role in alcohol control during the COVID-19 pandemic, ranging from direct lobbying advocacy, mass media advocacy and legal actions.

- **Direct lobbying advocacy**

During the COVID-19 pandemic, the Southern African Alcohol Policy Alliance -SAAPA- actively engaged in extensive advocacy related to COVID-19 alcohol restrictions. SAAPA is part of a broad alliance of CSOs working on advocacy in the Southern African region. SAAPA supported the bans or restrictions imposed on the sale and consumption of alcohol during the pandemic and advocated for continued restriction beyond the COVID-19 pandemic to avoid losing all ground gained while the restrictions were in place [17, 18]. SAAPA in South Africa supported these decisions because most people access alcohol where they live, especially from shebeens^1^ which are too small to implement physical distancing. In addition, binge drinking results in intoxication that impacts judgement which could lead to drinkers not following COVID-19 protective measures, including sharing drinking bottles and glasses. Finally, the government’s measures reduced the healthcare emergencies for trauma due to alcohol use [19]. Other than SAAPA, CSOs in South Africa reported focusing on preventing and managing gender-based violence and supporting the alcohol ban and restrictions.

SAAPA and its partners lobbied for additional tighter alcohol restrictions to minimize the risk of the spread of COVID-19. They petitioned the Government to restrict alcohol sales and advertising in South Africa and called for a host of restrictions to be placed on alcohol trade, including a ban on all alcohol advertising, restrictions on alcohol promotions and suspension of liquor licenses for establishments that contravened the Regulations, reduce off-consumptions operating hours, and temporarily impose zero breath and blood concentration levels for drivers [20]. They also used the momentum created by the alcohol bans in South Africa specifically to advocate for better long-term regulation of alcohol—advocating for the adoption of the Liquor Amendment Bill of 2016 in South Africa, which seeks to amend the Liquor Act of 2003 to better regulate distribution, trading and marketing of alcoholic beverages in alignment with the World Health Organisation Global Strategy for reducing the harmful use of alcohol and has been opposed by the alcohol industry [17, 21].

In Kenya, the NCD Alliance Kenya participated and made oral interventions during the Community COVID-19 Sub-committee meetings [22]. Additionally, CSOs in Namibia lobbied extensively for the adoption and enactment of the Draft National Alcohol Policy and disseminated information on the harm related to alcohol consumption [22].

b. **Public awareness media campaigns**

In July 2020, the NCD Alliance launched the Solidarity Fund on NCDs and COVID-19, totalling US$300000, to support 20 national and regional NCD alliances across all regions [23]. With the support of the Fund, alliances in Africa and other regions were able to step up their advocacy and communications efforts to promote the needs of people living with NCDs in national pandemic response plans.

SAAPA and its partners engaged in an education and awareness campaign through broadcasting, print and social media. SAAPA promoted a campaign tagged *new norm* advocating for evidence-based alcohol policies that reduce alcohol availability. They based their advocacy on the decline in alcohol-related harm seen when the sale and consumption of alcohol were restricted in the country in response to the COVID-19 pandemic [24]. SAAPA Lesotho hosted a press conference to launch the idea of a *new norm* and conducted several interviews on radio and television. SAAPA Lesotho was the first SAAPA chapter to advertise in a local newspaper to promote the idea of a *new norm*. They advocated for policy interventions to limit the amount of purchased alcohol, limit the hours and days of sale, the enforcement of license for trade, the enforcement of requesting identity cards for purchase of alcohol, for increasing the minimum age from 18 to 21, for no bulk buy specials, for increasing the price of alcoholic beverages, for restricting advertising at the point of sale, and for applying a lower blood-alcohol concentration limit while driving. Similar campaigns on the radio and in the local newspaper were carried out by SAAPA Zimbabwe [24].

On August 2020, the NCD Alliance Kenya urged the government to urgently address the underlying drivers of the NCD pandemic that fueled COVID-19 mortality and asked to prioritize the needs and care of People Living with NCDs by placing them at the centre of the COVID-19 response in Kenya [25].

Some CSOs reported building capacity amongst local volunteers through community training by educating them about the the health risks of alcohol use and the consequences of the harmful use of alcohol.

c. **Legal and regulatory interventions**

With Amandla.mobi, SAAPA South Africa launched a petition against the court challenges launched by alcohol manufacturers like South African Breweries and Vinpro [21]. SAAPA South Africa also initiated a complaint with the Press Council of South Africa concerning the SALBA partnership with the Sunday Times. The Deputy Press Ombud found that by assigning reporting staff to write two articles and treating these as conventional news stories, the Sunday Times had created the impression that these articles were independently produced. This complaint ultimately resulted in the Sunday Times publishing an apology for the content [26].

The alcohol ban and the public health justification put forward for the ban by the South African government were supported by the South African Medical Research Council (SAMRC) and other academics. They provided evidence of the reduced alcohol-related trauma caseload for the healthcare system to show the effectiveness of the alcohol ban [19, 27]. This was amplified by CSOs, including SAAPA and Movendi International, who used their platforms to share the evidence provided by the SAMRC and academics [28, 29].

These achievements are not without challenges, e.g., lack of resources and recognition. A study of West African CSOs working on alcohol harm prevention found that their work was adversely affected because of the pandemic including due to lack of adequate financial resources, and this may have affected access to services provided by CSOs in some countries [30].

The examples described in this paper illustrate the role CSOs can play in supporting government efforts further and contributing to alcohol control activities by generating evidence, documenting, monitoring and reporting on the implementation of alcohol control policies; advocating and engaging through various government technical working groups [31] such as alcohol, tobacco and drugs; lobbying for adopting evidence-based public health policies and National Alcohol Policy and Strategy in countries with no one; monitoring industry activities, conflict of interests and interference in public health policy-making; promoting a developmental and intersectional approach to alcohol control; assessing community needs in vulnerable settings; expanding the support for alcohol harm prevention in communities and specific groups; strengthening the delivery of services like treatment and rehabilitation services; promoting community-based education and awareness of the harm related to alcohol consumption; conducting awareness campaigns on prevention and treatment interventions.

For this, governments should create the conditions for CSOs to be empowered and engaged in public health policy development by recognizing the legal status of CSOs, providing resources and ensuring democratic CSO representation on statutory bodies and working groups.

## Conclusion

This paper sought to synthesize the role of CSOs in six African countries during the COVID-19 pandemic. Three areas were identified (i) direct lobbying advocacy, (ii) public awareness media campaigns and (iii) legal and regulatory interventions. Given the size of the challenges governments faced during the COVID-19 pandemic, the role of CSOs during the ongoing pandemic and beyond has become even more relevant for public health interventions aimed at reducing the harm derived from alcohol consumption in the studied countries. For this, CSOs should have a “seat at the table” when public health policies are designed, discussed and enforced.

## Data Availability

All data generated or analysed during this study are included in this published article.

## Review of actions led by CSO to reduce alcohol consumption during the COVID-19 pandemic

**Table:**

CSO key actions	Kenya (n = 1)	Lesotho (n = 7)	Namibia (n = 5)	South Africa (n = 4)	Tanzania (n = 1)	Zimbabwe (n = 1)
Direct lobbying advocacy to amplify and support government efforts to control alcohol consumption	Participated in and made oral submissions during the Community COVID-19 Sub-committee meetings	Lobbied for policy action including restrictions on purchasing amounts, limit hours and days of sale, and ID all sales of alcohol, among others Participated in government committee	Developed a petition to support a ban on advertisement and lobbied extensively for the adoption/enactment of the Draft National Alcohol Policy	Lobbyied for tighter alcohol restrictions including a ban on all alcohol advertising and restrictions on alcohol promotions; published articles and press releases to encourage the government’s efforts to control always use as a way of combatting COVID-19; encouraged the government to ignore pressure from the alcohol industry; interventions part of an Alliance Partner strategy Addressed the Ministerial Advisory Committee (MAC) headed by the Minister of Health, NEDLAC and other parliamentary bodies		
Conduct public awareness campaigns		Conducted a campaign tagged “New Norm” advocating for evidence-based alcohol policies that reduce alcohol availability	Shared information on harmful alcohol consumption Train the trainers to disseminate and educate communities on harmful alcohol consumption	Yes—launched the Solidarity Fund on NCDs and COVID-19 which stepped up advocacy and communications efforts to promote the needs of people living with NCDs in national pandemic response plans; writing evidence backed letters to the President, ministers. And National Coronavirus Command Centre; conduct press statements and interviews with media; protesting outside facilities where sale of alcohol was permitted; advocated for long term legislative measures to reduce alcohol-related harm	Yes	Carried out campaigns on the radio and in local newspapers to raise awareness for additional alcohol control policies
Launch legal challenges				Yes—launched legal challenges against alcohol manufacturers, as well as initiating a complaint with the Press Council concerning the partnership between industry and the press		

## Footnotes

1 These are illegal outlets, often someone's home. The name originates from Ireland and historically associated with illegal supply of alcohol.
